# Acute Pancreatitis in Pregnancy and the Early Postpartum Period: An Anaesthesiology and Critical Care Perspective

**DOI:** 10.3390/jcm15082968

**Published:** 2026-04-14

**Authors:** Krisztina Tóth, Zsombor Márton, Csaba Csontos, Sándor Márton

**Affiliations:** 1 Department of Anaesthesiology and Intensive Therapy, University of Pécs Medical School, 7624 Pécs, Hungary; toth.krisztina2@pte.hu (K.T.); csontos.csaba@pte.hu (C.C.); 2 Department of Pediatric Surgery, University of Pécs Medical School, 7624 Pécs, Hungary; marton.zsombor@pte.hu

**Keywords:** acute pancreatitis, pregnancy, postpartum period, obstetric anaesthesia, critical care, epidural analgesia, maternal morbidity

## Abstract

**Background/Objectives:** Acute pancreatitis in pregnancy and the early postpartum period (APIP) is an uncommon but potentially life-threatening condition associated with significant maternal morbidity. Physiological adaptations of pregnancy, recent obstetric surgery, and overlapping postoperative symptoms frequently obscure early diagnosis and complicate perioperative and critical care management. This review provides a clinically oriented, anaesthesiology-focused overview of APIP, integrating current evidence with perioperative decision-making, pain management strategies, and intensive care considerations relevant to obstetric practice. **Methods:** A narrative, clinically structured review of the literature was performed focusing on epidemiology, aetiology, diagnosis, severity stratification, and management of APIP. Anaesthesiology- and ICU-specific aspects are synthesised into a pragmatic management framework. **Results:** Gallstone disease and hypertriglyceridaemia remain the predominant causes of APIP, with most cases occurring in the third trimester or early postpartum period. Diagnosis relies on pancreatic enzyme elevation and pregnancy-adapted imaging strategies. Early goal-directed fluid resuscitation, effective multimodal analgesia, and timely initiation of enteral nutrition are key determinants of outcome. Therapeutic ERCP and laparoscopic cholecystectomy can be safely performed during pregnancy when clinically indicated and may reduce recurrence in biliary pancreatitis. Neuraxial analgesia provides effective, opioid-sparing pain control and may improve respiratory mechanics and haemodynamic stability. Persistent organ failure remains the strongest predictor of adverse outcome and should prompt early intensive care admission. **Conclusions:** APIP requires early recognition and severity-adapted, multidisciplinary management. Anaesthesiology-led strategies play a central role in optimising analgesia, haemodynamic stability, and timely escalation of care. Framing APIP within a perioperative and critical care context may improve maternal outcomes in this vulnerable patient population.

## 1. Introduction

Acute pancreatitis during pregnancy and the early postpartum period is a rare but clinically important condition, with an estimated incidence of 1–3 cases per 10,000 pregnancies [1,2]. Despite its low frequency, moderate to severe disease may be associated with substantial maternal morbidity and, in selected cases, foetal compromise [3]. Historically, maternal and foetal mortality rates were reported to be as high as 30–60%; however, advances in diagnostic imaging, intensive care, and multidisciplinary management have markedly improved outcomes over recent decades [1,3].

The temporal distribution of APIP is characteristic, with most cases occurring in the third trimester or in the early postpartum period [1,3]. During this phase, abdominal pain, nausea, vomiting, and ileus may overlap with obstetric, surgical, or postoperative conditions, particularly following caesarean delivery, increasing the risk of delayed diagnosis.

From an obstetric anaesthesia perspective, APIP represents a challenging perioperative condition that often requires early anaesthesiology involvement for haemodynamic stabilisation, analgesia, and critical care triage. Pregnancy-related cardiovascular, respiratory, and metabolic adaptations modify the systemic inflammatory response and may mask early organ dysfunction. Anaesthesiologists are frequently involved in analgesia, haemodynamic stabilisation, airway management, and ICU triage; nevertheless, these aspects remain underrepresented in many reviews focusing primarily on obstetric or gastroenterological perspectives. An anaesthesiology-centred narrative is therefore clinically relevant.

## 2. Methods

### 2.1. Study Design and Scope

No specific instruments, proprietary software, or commercial agents requiring manufacturer identification were used in this study.

The manuscript is based on a narrative synthesis of previously published literature. Standard bibliographic databases (PubMed, MEDLINE, Embase) were used for literature retrieval; these are publicly accessible platforms and do not have fixed version numbers.

Therefore, company names, addresses, and software version numbers are not applicable.

### 2.2. Literature Identification and Selection

Relevant literature was identified through targeted searches of the PubMed, MEDLINE, and Embase databases, focusing on publications addressing acute pancreatitis in pregnancy, postpartum pancreatitis, obstetric anaesthesia, pain management, and intensive care management. Priority was given to clinical studies, reviews, consensus guidelines, and landmark articles published in peer-reviewed journals. The reference lists of key articles were also manually screened to identify additional relevant sources.

### 2.3. Data Synthesis and Clinical Framework

The retrieved evidence was synthesised thematically, with emphasis on epidemiology, aetiology, diagnostic strategies, severity assessment, and perioperative management. Anaesthesiology- and ICU-specific considerations—including analgesic strategies, fluid resuscitation, haemodynamic monitoring, respiratory support, and criteria for intensive care admission—were integrated into a pragmatic, severity-adapted management framework. The available evidence was interpreted in the context of pregnancy-related physiological adaptations and postpartum surgical stress.

### 2.4. Ethical Considerations

This article is based on previously published data and anonymised clinical experience. No new experimental interventions were performed; therefore, formal ethical approval was not required.

## 3. Aetiology and Pathophysiology

Gallstone disease is the most common cause of acute pancreatitis during pregnancy, accounting for more than half of reported cases [4,5]. Hormonal influences, particularly progesterone, alter bile composition and gallbladder motility, promoting biliary stasis and gallstone formation. These mechanisms are most pronounced in late pregnancy, corresponding with the peak incidence of APIP [5]. Hypertriglyceridaemia represents the second most frequent aetiology and is associated with a higher likelihood of severe disease [2,6]. Pregnancy-related metabolic changes may increase plasma triglyceride levels two- to threefold due to reduced lipoprotein lipase activity and enhanced hepatic lipid synthesis. The risk of pancreatitis rises substantially when triglyceride levels exceed 500 mg/dL, particularly in patients with obesity or pre-existing dyslipidaemia [6]. 

Less common causes include drug-induced pancreatitis, alcohol consumption, and idiopathic disease. In the early postpartum period, surgical stress and inflammatory activation following caesarean delivery may contribute to disease onset in selected cases [7,8]. Regardless of aetiology, premature pancreatic enzyme activation initiates a local inflammatory cascade that may progress to systemic inflammatory response syndrome and multiorgan failure. Systemic inflammatory response and mediator activation play a key role in disease progression [9,10,11].

## 4. Diagnosis and Imaging

Diagnosis of acute pancreatitis in pregnancy relies on the same core criteria as in the non-pregnant population [12,13,14]. Clinical presentation, however, may be atypical, particularly in late gestation or in the immediate postoperative period following caesarean delivery. Differential diagnostic considerations are particularly important in pregnant and early postpartum patients, where multiple obstetric and non-obstetric conditions may mimic acute pancreatitis. Key distinguishing features are summarised in Table 1.

## 5. Severity Assessment and Prognosis

Severity stratification is a cornerstone of clinical decision-making in acute pancreatitis, guiding monitoring intensity, level of care, and therapeutic strategy. Severity classification and complication definitions are based on the Revised Atlanta Classification, which is summarised in Table 2 [12].

## 6. Perioperative and Intensive Care Management Algorithm in APIP

Acute pancreatitis in pregnancy and the early postpartum period (APIP) requires structured, severity-adapted perioperative and critical care management. Pregnancy-related physiological changes and the frequent overlap with obstetric or postoperative conditions may delay diagnosis and obscure early organ dysfunction. Anaesthesiologists therefore play a central role in early triage, haemodynamic stabilisation, analgesia, respiratory support, and the timely escalation of care.

### 6.1. Initial Assessment and Triage

Early clinical assessment should focus on identifying systemic inflammatory response and early organ dysfunction. Particular attention should be paid to haemodynamic instability, hypoxaemia, oliguria, and altered mental status, which may be masked by pregnancy- and postpartum-related physiological adaptations.

Laboratory evaluation should include pancreatic enzymes, inflammatory markers, renal and hepatic function tests, and serum triglyceride levels. Severity stratification should be performed early using the Revised Atlanta Classification [12].

Given the potential for rapid deterioration, a lower threshold for high-dependency or intensive care unit (ICU) admission is recommended in pregnant and postpartum patients, particularly in the presence of persistent systemic inflammatory response, respiratory compromise, or increasing analgesic requirements.

### 6.2. Fluid Resuscitation and Haemodynamic Management

Balanced crystalloids, particularly lactated Ringer’s solution, are preferred over normal saline due to their favourable effects on acid–base balance and systemic inflammation [15].

Haemodynamic targets should be individualised, with close monitoring of mean arterial pressure, urine output, serum lactate, and dynamic indices of fluid responsiveness when available. Invasive arterial blood pressure monitoring should be considered in patients with haemodynamic instability or severe disease. Vasopressor support may be required in refractory hypotension and should be initiated early in an ICU setting.

### 6.3. Analgesia and Pain Management

Effective pain control is essential in APIP management. Multimodal analgesia strategies are recommended, combining paracetamol, carefully titrated opioids, and neuraxial techniques when appropriate. Epidural analgesia may provide opioid-sparing pain relief, improve respiratory mechanics, and support haemodynamic stability.

### 6.4. Respiratory Support and ICU Admission Criteria

Respiratory compromise may develop early due to pain-related hypoventilation, systemic inflammation, or fluid overload. Continuous pulse oximetry and arterial blood gas analysis should be used liberally. Supplemental oxygen should be initiated promptly, and non-invasive ventilation may be considered in selected cases.

Indications for ICU admission include persistent organ failure lasting longer than 48 h, escalating oxygen requirements, haemodynamic instability, acute kidney injury, or suspected infected pancreatic necrosis. Early multidisciplinary involvement—including anaesthesiology, intensive care, obstetrics, gastroenterology, and surgery—is essential to optimise maternal outcomes.

### 6.5. Postpartum-Specific Considerations

Analgesic strategies must consider breastfeeding safety, while nutritional planning should support maternal recovery and prevent metabolic deterioration. Early mobilisation, thromboprophylaxis, and vigilant monitoring for postoperative complications are integral components of care.

### 6.6. Interventional and Surgical Management During Pregnancy

Biliary pancreatitis represents the most frequent aetiological subtype of APIP, and definitive management of the underlying cause is often required to prevent recurrence. Endoscopic retrograde cholangiopancreatography (ERCP) with sphincterotomy is considered safe during pregnancy when performed by experienced teams with minimised fluoroscopy time and appropriate foetal shielding. ERCP is primarily indicated in cases of persistent biliary obstruction, cholangitis, or ongoing clinical deterioration despite conservative management. Available evidence suggests that therapeutic ERCP during pregnancy is associated with favourable maternal outcomes and low foetal complication rates when radiation exposure is carefully controlled [16].

Laparoscopic cholecystectomy is regarded as the definitive treatment for gallstone-related pancreatitis. Current data indicate that laparoscopic surgery can be performed safely during all trimesters, although the second trimester has traditionally been considered optimal due to lower risks of miscarriage and preterm labour. Early cholecystectomy following mild biliary pancreatitis may reduce recurrence risk and unplanned hospital readmissions.

In cases of severe pancreatitis with persistent organ failure or necrotising disease, surgical or minimally invasive interventions should follow standard severity-based principles. Timing of intervention should be individualised and ideally postponed until the necrotic collection has become walled-off, unless sepsis or abdominal compartment syndrome mandates earlier action. Multidisciplinary coordination between obstetrics, surgery, gastroenterology, anaesthesiology, and intensive care teams is essential to optimise maternal and foetal safety.

Key anaesthesiology- and intensive-care-specific considerations related to interventional and perioperative management are summarised in Table 3.

## 7. Discussion

Recent predictive models, including nomogram-based approaches, have been proposed to improve early risk stratification in acute pancreatitis during pregnancy, although their clinical applicability remains to be validated in larger cohorts [17]. A previously published postpartum case from our institution further illustrates the diagnostic challenges of acute pancreatitis following caesarean delivery [8]. Building on this clinical observation, acute pancreatitis in pregnancy and the early postpartum period (APIP) represents a uniquely complex clinical entity situated at the intersection of obstetric medicine, perioperative care, and critical illness. Although epidemiologically rare, APIP carries disproportionate clinical significance because of its diagnostic ambiguity, its potential for rapid deterioration, and the physiological vulnerability imposed by pregnancy-related adaptations. One of the central challenges in APIP is diagnostic delay, which frequently arises from symptom overlap with obstetric, surgical, and postoperative conditions. Abdominal pain, nausea, ileus, and haemodynamic variability are common in late pregnancy and following caesarean delivery, often obscuring early recognition of pancreatic inflammation. This diagnostic uncertainty is not merely a logistical concern but a pathophysiological hazard, as delayed diagnosis is strongly associated with increased disease severity, higher rates of organ failure, and prolonged maternal morbidity. The present review underscores that clinical vigilance and early biochemical evaluation remain critical determinants of outcome. The Revised Atlanta Classification [12] (Banks et al., 2013) provides an essential framework for severity stratification; however, its application in pregnant patients warrants nuanced interpretation. Pregnancy induces profound cardiovascular, respiratory, and renal adaptations that may mask early indicators of systemic deterioration. Increased cardiac output, reduced systemic vascular resistance, and altered intravascular volume dynamics may transiently preserve macrocirculatory parameters despite evolving microcirculatory dysfunction. Similarly, reduced functional residual capacity and pregnancy-associated respiratory mechanics may accelerate hypoxaemia once pulmonary complications develop. Consequently, organ failure in APIP may manifest abruptly rather than progressively, supporting the rationale for a lower threshold for high-dependency monitoring and ICU admission. The present review provides an anaesthesiology-centred framework that integrates perioperative risk assessment, multimodal analgesia strategies, and critical care escalation pathways for pregnant and postpartum patients with acute pancreatitis. 

## 8. Conclusions

Acute pancreatitis in pregnancy and the early postpartum period remains a rare but clinically significant condition requiring heightened clinical awareness, particularly in the context of overlapping obstetric and postoperative presentations. Early recognition, timely severity stratification, and pregnancy-adapted diagnostic strategies are critical to prevent delayed intervention and progression to organ failure. Severity-adapted, multidisciplinary management represents the cornerstone of care, with anaesthesiology-led strategies playing a pivotal role in optimising analgesia, haemodynamic stability, respiratory support, and timely escalation to intensive care. Structured perioperative decision-making and early involvement of critical care teams may mitigate maternal morbidity and improve short-term outcomes. Close collaboration between obstetricians, anaesthesiologists, intensivists, gastroenterologists, and surgeons is essential to ensure coordinated care throughout pregnancy and the postpartum period. Future studies should focus on refining risk stratification tools and developing pregnancy-specific management pathways to further improve maternal safety in this vulnerable patient population.

## Figures and Tables

**Table 1:** Differential diagnostic considerations in pregnant and early postpartum patients presenting with acute abdominal pain.

Condition	Key Clinical Features	Laboratory Findings	Distinguishing Characteristics
Acute pancreatitis	Epigastric or upper abdominal pain radiating to the back, nausea, vomiting	Elevated amylase and/or lipase (>3× ULN), possible hypertriglyceridaemia	Imaging may show pancreatic inflammation; pain often severe and persistent
HELLP syndrome	Right upper quadrant or epigastric pain, hypertension, malaise	Elevated liver enzymes, thrombocytopenia, haemolysis markers	Associated with preeclampsia; pancreatic enzymes typically normal
Acute fatty liver of pregnancy (AFLP)	Nausea, vomiting, abdominal pain, jaundice, encephalopathy (late)	Hypoglycaemia, elevated liver enzymes, coagulopathy	Rapid hepatic dysfunction; may mimic severe systemic illness
Biliary colic/cholecystitis	Right upper quadrant pain, often postprandial	Mild liver enzyme elevation, normal pancreatic enzymes (unless complicated)	Ultrasound detects gallstones; pain episodic (colic)
Intestinal obstruction/ileus	Diffuse abdominal pain, distension, vomiting	Non-specific; possible electrolyte imbalance	Imaging shows bowel dilation; absence of pancreatic enzyme elevation
Appendicitis	Migrating abdominal pain, fever, localised tenderness	Elevated inflammatory markers	Location may shift in pregnancy; pancreatic enzymes normal
Postoperative complications	Localised pain, fever, haemodynamic instability	Variable depending on cause	Context of recent surgery; imaging clarifies diagnosis

ULN: upper limit of normal.

**Table 2:** Revised Atlanta classification of acute pancreatitis.

Category	Definition/Criteria	Clinical Implications
Disease Types		
Interstitial edematous pancreatitis	Pancreatic enlargement due to inflammatory oedema without necrosis	Typically associated with mild disease
Necrotising pancreatitis	Pancreatic and/or peripancreatic necrosis	Higher risk of organ failure and complications
Severity Classification		
Mild acute pancreatitis	No organ failure; no local or systemic complications	Usually self-limiting
Moderately severe acute pancreatitis	Transient organ failure (<48 h) and/or local or systemic complications	Intermediate clinical course
*Severe acute pancreatitis*	Persistent organ failure (>48 h)	High morbidity and mortality
*Organ Failure*		
Organ failure	Defined using the modified Marshall scoring system	Determines severity
Transient organ failure	Resolves within 48 h	Associated with moderately severe disease
Persistent organ failure	Lasts > 48 h	Defines severe pancreatitis
Local Complications		
Acute peripancreatic fluid collection (APFC)	Early fluid collection without a defined wall	Often resolves spontaneously
Pancreatic pseudocyst	Encapsulated fluid collection (>4 weeks) without necrotic content	Typically benign course
Acute necrotic collection (ANC)	Early necrotic collection	May evolve into walled-off necrosis
Walled-off necrosis (WON)	Encapsulated necrosis (>4 weeks)	May require intervention

Adapted from the Revised Atlanta Classification (Banks et al., 2013) [12].

**Table 3:** Anaesthesiology- and intensive-care-specific management considerations in APIP.

Domain	Pathophysiological Considerations	Clinical Implications
Initial assessment	Risk of masked early organ dysfunction due to pregnancy-related adaptations	Lower threshold for HDU/ICU monitoring
Fluid therapy	Risk of hypoperfusion vs. pulmonary oedema	Goal-directed resuscitation using balanced crystalloids
Haemodynamic monitoring	Altered cardiovascular physiology	Consider invasive arterial monitoring in unstable patients
Analgesia	Sympathetic activation, impaired ventilation	Multimodal, opioid-sparing; neuraxial techniques preferred
Respiratory care	Reduced FRC and pain-related splinting	Early oxygen supplementation and ABG monitoring
ICU triggers	Persistent organ failure > 48 h	Early ICU transfer
Postpartum considerations	Surgical stress, lactation, hypercoagulability	Breastfeeding-compatible analgesia; thromboprophylaxis

## Data Availability

No new data were created or analysed in this study.
